# Chronic Granular Conjunctivitis, or Trachoma

**Published:** 1888-03

**Authors:** F. E. Yoakum

**Affiliations:** President; Greenville, Texas


### CHRONIC GRANULAR CONJUCTIVITIS OR TRACHOMA.

Read Before the East Line Texas Medical Association, Feb., 7th. 1888,

*By F. E. Yoakum, M. D. President.*

For Daniel's Texas Medical Jouanal.

I endeavor to present a short paper on one of the most important subjects if not the most vexed theme as general practitioners or specialist, we are called upon to treat every day in our work. The strides that have been made in this most troublesome disease in the last three or four years is most remarkable, both the etiology and treatment. I will give in this short paper the different treatments of a few of the leading oculists, as I have seen it applied. Dr. R. uses the crystals of muriate of ammonia, applied to the inverted lids very firmly and evenly to the upper and lower lids. The pain is very great at first, but only lasts for a few seconds, when it leaves the eye clear and pleasant with moisture. (This treatment I use a great deal.) This is applied every day or two, and if the pain should continue five minutes he uses cocaine, a treatment used with wonderful success. Prof. M. uses jequerity very extensively, and especially when there is dizziness and inability to raise the lids, a treatment which connot be excelled in a great many trachomatous cases. The aqueous solution is dropped into the eye, then the lids are closed and well rubbed for several seconds with the fingers. This remedy is repeated every three or four weeks, that is, when the jequeritous inflammation has subsided entirely. With this treatment it is necessary to be very watchful and see the patient every day until the inflammation begins to subside. Dr. H., in the mildest forms uses the crystal of sulph cupri to the inverted eylids, applying it very carefully to the retrotarsal folds first, then touching every part of the lids, when he thoroughly washes out the excess with clear water, using a large camel hair pencil, then instilled a few drops of a solution of cocaine. This treatment in some cases was repeated every day and in others every two days. He believes that success will follow this treatment in almost every case of the mild form of trachoma; this plan, and perfect cleanliness is strictly adhered to. Now in the well pronounced exagerated cases his treatment is quite different, and one peculiarly his own. He has applied it for five years. And since publication of this plan the leading men of the world have used it successfully. Upon the upper eyelid it is usually executed in this way: While the thumb or forefinger of the left hand are holding the lid inverted he inserts the end of the thumb or forefiuger of the right hand, with the palmer side turned toward the eye-ball under the inverted lid. Thus the upper tarsal border and the swollen or infiltrated, retrotarsal fold are placed just between the thumbs (or forefingers) and if now these fingers are pushed toward each other, and at the same time the thumb (or forefinger) of fhe right hand is made to glide slowly forward from under the trachomatous conjuctival fold, this is subjected to a steady and continuous pressure, which will force out the contents of the trachoma follicles. By repeating this manipulation several times, if necessary, along the whole breadth of the eyelid, you can in one treatment remove all the trachoma follicles. Instead of the fingers we might, of course use a spatula or like instrument, He prefers using the fingers, because they are always at hand, and because, he can estimate the amount of pressure better with fingers than with an instrument. Now this is almost his exact desc·iption of his plan. I saw it used this winter in Chicago, and in the majority of the cases it acted like a charm, The squeezing process is sometimes very painful, and even with cocaine the patients cry out with pain. This after a day or two is followed with a weak solution of sublimate (gr ¼ to aq. 1 oz) or when there was considerable catarrhal secretion following use a solution of nitrate of silver (gr. v to x to 1 oz) used with camels hair pencil. (I have the treat-ment of other noted professors, which I will report in the future, and will refrain for fear of wearying you and your readers.

Respectfully,

F. E. YOAKUM, M. D.

